# Bacterial Composition Associated With Giant Colonies of the Harmful Algal Species *Phaeocystis globosa*

**DOI:** 10.3389/fmicb.2021.737484

**Published:** 2021-09-17

**Authors:** Zhu Zhu, Rui Meng, Walker O. Smith Jr., Hai Doan-Nhu, Lam Nguyen-Ngoc, Xinjun Jiang

**Affiliations:** ^1^ School of Oceanography, Shanghai Jiao Tong University, Shanghai, China; ^2^ Vietnam Academy of Science and Technology, Institute of Oceanography, Nha Trang, Vietnam

**Keywords:** bacterial consortia, colony, colonial envelope, 16S rRNA, microenvironment, *Phaeocystis globosa*

## Abstract

The cosmopolitan algae *Phaeocystis globosa* forms harmful algal blooms frequently in a number of tropical and subtropical coastal regions in the past two decades. During the bloom, the giant colony, which is formed by *P. globosa*, is the dominant morphotype. However, the microenvironment and the microbial composition in the intracolonial fluid are poorly understood. Here, we used high-throughput 16S rRNA amplicon sequencing to examine the bacterial composition and predicted functions in intracolonial fluid. Compared with the bacterial consortia in ambient seawater, intracolonial fluids possessed the lower levels of microbial richness and diversity, implying selectivity of bacteria by the unique intracolonial microenvironment enclosed within the *P. globosa* polysaccharide envelope. The bacterial consortia in intracolonial fluid were dominated by *Balneola* (48.6% of total abundance) and *Labrezia* (28.5%). The bacteria and microbial function enriched in intracolonial fluid were involved in aromatic benzenoid compounds degradation, DMSP and DMS production and consumption, and antibacterial compounds synthesis. We suggest that the *P. globosa* colonial envelope allows for the formation of a specific microenvironment; thus, the unique microbial consortia inhabiting intracolonial fluid has close interaction with *P. globosa* cells, which may benefit colony development.

## Introduction

*Phaeocystis globosa* is a cosmopolitan marine phytoplankton that plays important roles in carbon and sulfur biogeochemical cycles (Schoemann et al., 2005). *P. globosa* forms large blooms and is considered to be a harmful algal bloom species due to its negative economic and ecological impacts. Blooms of *P. globosa* in temperate regions, especially in the North Sea, have been observed regularly since the 1970s and are associated with nutrient enrichment from rivers (Cadee and Hegeman, 2002). Outbreaks of *P. globosa* blooms have been reported in tropical and subtropical regions frequently in the past two decades, including coastal waters of China (Qi et al., 2004), Viet Nam (Tang et al., 2004; Doan-Nhu et al., 2010; Smith et al., 2014) and the Arabian Sea (Madhupratap et al., 2000).

A unique feature of *Phaeocystis* is its polymorphic life cycle, which includes free-living solitary cells and colonies. A colony is balloon-like, with an envelope consisting of mucilaginous polysaccharides excreted by cells that are in turn embedded in this envelope (Baumann et al., 1994; Vanrijssel et al., 1997). The interior of the colony is filled with seawater. *P. globosa* colonies usually reach several millimeters in diameter; however, observations in Asian waters have shown that colonies can reach 1cm, with the maximum diameter being *ca.* 3cm (Qi et al., 2004). Individual cells (both those embedded in colonies and solitary forms) are only 3–12.4μm in diameter (Rousseau et al., 1994; Smith et al., 2014). Colony formation may provide a number of competitive advantages relative to free-living solitary cells. For example, the large size protects colonial cells from being grazed by small zooplankton due to size mismatch (Jakobsen and Tang, 2002). Additionally, the polysaccharide envelope may physically protect colonial cells against infection from bacteria and viruses (Hamm, 2000). Numerous studies have shown that colonies are the dominant forms in *P. globosa* blooms (Qi et al., 2004; Smith et al., 2014), supporting the perspective that *Phaeocystis* blooms are linked to the ecological advantages of colony formation. It remains unclear why the sizes of Asian *Phaeocystis* colonies are greater than those found in other areas of the ocean.

*Phaeocystis* colonies have special characteristics imparted by the envelope. The envelope is a thin yet mechanically stable skin (Hamm et al., 1999), forming a diffusive boundary layer that may limit nutrient and solute fluxes across the envelope (Ploug et al., 1999). The interior liquid develops into a unique microenvironment that is significantly different from ambient seawater. Smith et al. (2014) demonstrated that dissolved organic carbon (DOC) concentrations of intracolonial fluid were 25 times greater than ambient concentrations. Furthermore, the pH inside young healthy colonies can exceed those of ambient seawater (Lubbers et al., 1990), suggesting the flux of some ions is restricted, a finding that direct experiments verified (Smith et al., 2014).

The microenvironment provided by phytoplankton offers microscale niches for its associated heterotrophic bacteria (Seymour et al., 2017). For example, in the *Trichodesmium* holobiont, *Trichodesmium* colonies create a chemically heterogeneous microenvironment (Klawonn et al., 2020), presumably supporting diverse microbial composition and metabolisms, including phosphate and iron acquisition and denitrification pathways in millimeter-sized colonies (Gradoville et al., 2017), which in turn facilitate growth of *Trichodesmium*. The unique intracolonial microenvironment of *P. globosa* may harbor specific microbial consortia, which in turn facilitate the growth of *P. globosa* colonies. However, the microbial diversity and composition of the intracolonial microenvironment is unknown.

In the Amundsen Sea, it has been shown that microbial diversity within *P. antarctica* blooms was greatly reduced (Delmont et al., 2014), and the dominant microbes had metabolic features that were tied to the metabolism of *Phaeocystis* and sulfur cycling (Delmont et al., 2015). A number of bacteria in ambient seawater were clearly associated with *Phaeocystis*, and it was suggested that the bacteria and *Phaeocystis* established a mutualistic association (Delmont et al., 2014). In the northern Beibu Gulf of China, Xu et al., 2021 demonstrated that microbial communities from *P. globosa* bloom and non-bloom sites exhibited distinct community composition, and the bloom microbes were likely driven by high environmental selection. However, the microbial community enclosed by the colony envelope is still unknown, and hence the importance of the microenvironment in establishing the unique phytoplankton-bacteria association remains unclear.

We sampled the internal fluid of giant *P. globosa* colonies collected in Vietnamese coastal waters and analyzed its bacterial composition, sequenced 16S rRNA amplicons, and compared them to those found in ambient seawater to test if the intracolonial microenvironment of *P. globosa* provides a unique habitat for the development of a specific microbial assemblage. Our results suggest that the microenvironment is indeed different and leads to the development of a microbial consortia that is distinct from the ambient environment.

## Materials and Methods

### Sample Collection

**Figure 1:**
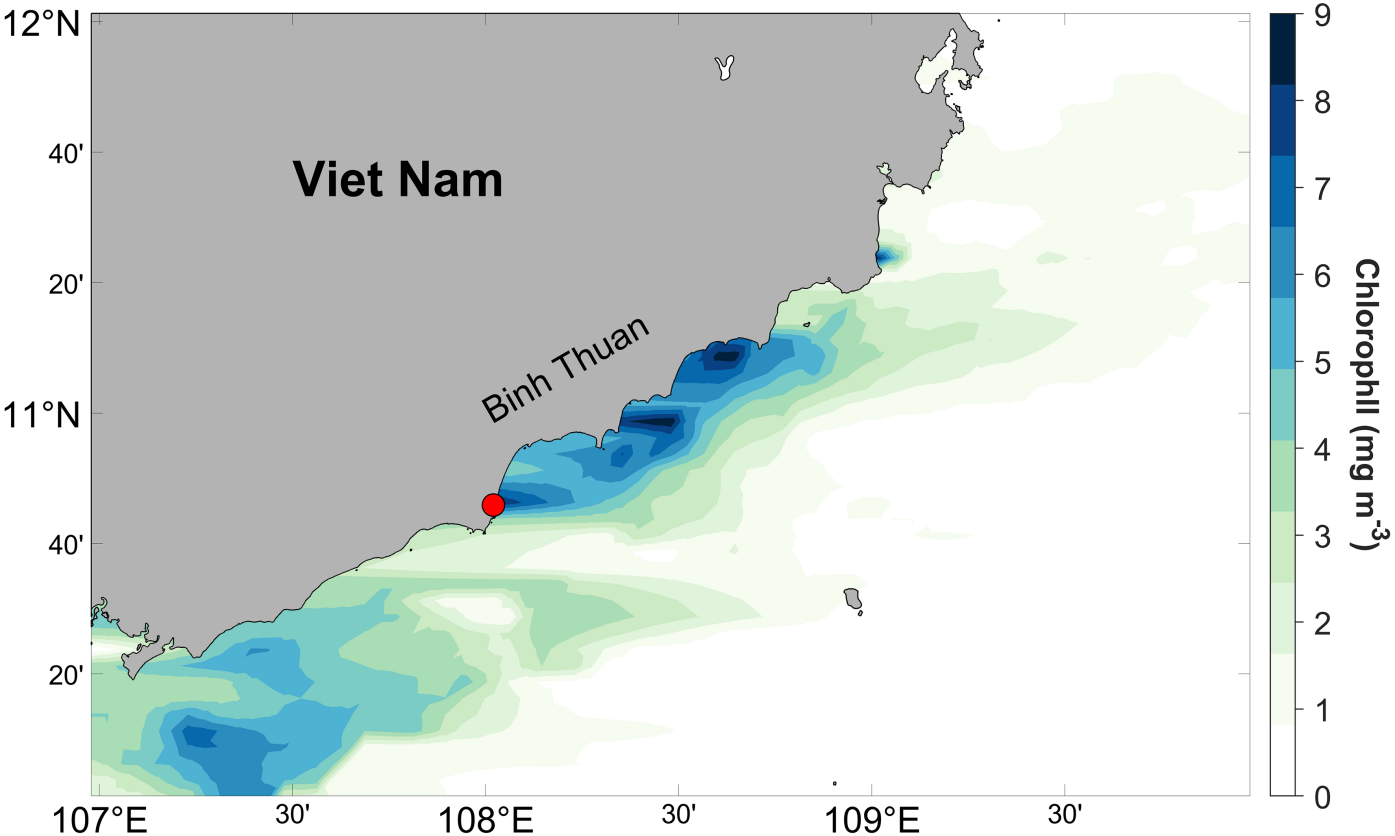
Monthly averaged chlorophyll a concentration off the southcentral Viet Nam derived from MODIS-Aqua satellite estimates for August 2019. The red circle represents the sampling location.

Samples during a *P. globosa* giant colony bloom were collected in August 2019 off the coast of Phan Thiet, southcentral Viet Nam (Figure 1), where blooms regularly occur during summer (Tang et al., 2004; Doan-Nhu et al., 2010). Chlorophyll a data for August 2019 were obtained from the Moderate Resolution Imaging Spectroradiometer aboard the Aqua (MODIS-Aqua) satellite with the horizontal resolution of 4km, and the data were extracted and binned from 10–12°N, 107–100°E to generate the monthly averaged chlorophyll a concentration along the coast. In addition to abundant intact spherical colonies, there were substantial numbers of collapsed colonies in the coastal zone, likely due to extreme turbulence of the near-shore waters during sampling. The maximum size of *P. globosa* colonies we observed was *ca.* 1.5cm, while diameters of most intact colonies were 0.4–1.0cm. Large volume (~10L) of surface seawater was collected, 500–1,000ml seawater was used for collecting seawater bacteria consortia, and the remaining seawater was used for picking *P. globosa* colonies. Hand-picked intact colonies were kept in a smaller container (~1L) filled with seawater for subsequent procedure.

Freshly collected *P. globosa* colonies were used to sample the internal microenvironment. First, a single colony was transferred with a small plastic spoon into a Petri dish for diameter measurement, after which the colony was immediately transferred to a new container containing similar-size colonies. We chose 0.7–1cm-diameter colonies for intracolonial fluid collection, as smaller colonies were more difficult to extract and provided a smaller amount of internal fluid for analysis. Internal fluids were directly extracted using a sterile syringe fitted with small-gauge needle. About 7ml of pooled intracolonial fluid, collected from *ca.* 20 colonies, was filtered through sterile 0.2-μm polycarbonate filters (Merck Millipore) to collect intracolonial bacteria. Seawater samples were pre-filtered through Whatman 47mm GF/F filters (nominal pore size ~0.7μm) to remove *P. globosa* colonies and larger particles. Then, sterile 0.2-μm pore-size polycarbonate filters were used to collected seawater bacteria. All filters were preserved in 2ml RNAlater solution (Life Technologies, Gaithersburg, MD, USA) and stored at −20°C until further processing. Three replicates were collected for intracolonial fluid samples and seawater samples, respectively.

### DNA Extraction and 16S rRNA Sequencing

Microbial DNA was extracted from filters by employing FastDNA Spin Kit for Soil (MP Biomedicals, Heidelberg, Germany) according to manufacturer’s instructions. Primer pair 338F (5'-ACTCCTACGGGAGGCAGCAG-3') and 806R (5'-GGACTACHVGGGTWTCTAAT-3') was first chosen to amplify V3-V4 region of the bacterial 16S rRNA gene. However, initial results suggested substantial chloroplast contamination. To minimize chloroplast contamination, primer pair 799F (5'-AACMGGATTAGATACCCKG-3') and 1193R (5'-ACGTCATCCCCACCTTCC-3'; Beckers et al., 2016), which amplified the V5-V7 region of 16S rRNA gene, was subsequently used.

A nested PCR strategy was performed to amplify all samples and thereby minimize the formation of primer dimers according to Beckers et al. (2016). A first round of PCR amplification was conducted using primer pair 799F and 1392R (5'-GACGGGCGGTGWGTRCA-3') by an ABI GeneAmp^®^ 9700 PCR thermocycler (Applied Biosystems, Foster City, CA, USA). Each 20μl PCR contained 10ng of template DNA, 1U *TransStart* FastPfu DNA Polymerase (TransGen, Beijing, China), and a final concentration of 1×*TransStart* FastPfu buffer, 0.25mm dNTPs and 0.2μm of each primer. The PCR amplification consisted of a 3-min initial denaturation step at 95°C, followed by 27 cycles each at 95°C for 30s, 55°C for 30s and 72°C for 45s, and final extension at 72°C for 10min. PCR products were purified with Agencourt AMPure XP Beads (Beckman Coulter, Brea, CA, USA) and used as the second-round PCR templates. A second round of PCR amplification was carried out in triplicate for primer pair of 799F-1193R with Illumina adapters and the sample-specific barcodes. PCR were identical as the first-round PCR, with the exception of only using 13 PCR cycles this time. The second-round PCR products were subsequently purified using the AxyPrep DNA Gel Extraction Kit (Axygen Biosciences, Union City, CA, USA), and the quality of the amplicons was evaluated using Quantus^™^ Fluorometer (Promega, Madison, WI, USA) according to manufacturer’s instructions.

Purified amplicons were pooled in equimolar and paired-end sequenced on an Illumina MiSeq PE300 platform (Illumina, San Diego, CA, USA) according to the standard protocols by Majorbio Bio-Pharm Technology Co. Ltd. (Shanghai, China). The raw reads were deposited into the NCBI Sequence Read Archive (SRA) database and are available under the accession number: PRJNA720571.

### 16S rRNA Sequence Analyses

The raw 16S rRNA gene sequencing reads were demultiplexed, quality-filtered by Fastp version 0.20.0 (Chen et al., 2018) and the paired-end reads were merged by FLASH version 1.2.7 (Magoc and Salzberg, 2011). Quality-filtered sequences were clustered into operational taxonomic units (OTUs) with 97% similarity cutoff using UPARSE v.7.1 (Edgar, 2013). Chimeric sequences were identified and removed. The taxonomies of representative OTU (the most abundant sequence in each OTU) were classified by RDP Classifier (Wang et al., 2007) against the Silva database version 138. Sequences matching “Chloroplast” and “Mitochondria” were moved. To correct for differences in sequencing depth, OTU counts were rarefied to the lowest number of sequences per sample (13,172 sequences) and OTUs with fewer than 15 counts across all the samples were excluded. The final OTU table is provided as Supplementary Table S1.

Alpha-diversity of each sample was assessed by community richness or OTU richness estimator (Chao 1 estimator) and community diversity estimator (Shannon’s diversity index). Statistical difference in alpha-diversity between the microbial assemblage of seawater and intracolonial fluid was calculated using a Student’s *t* test. The significance level was *a priori* set at 0.05.

The top 50 most abundant OTUs were used for taxa analysis. Linear discriminant analysis (LDA) effect size (LEfSe) algorithm (Segata et al., 2011) was applied through the Galaxy server to identify significant differentially abundant bacterial taxa enriched in the seawater or intracolonial fluid. Only those OTUs whose LDA score > 2 and value of *p*<0.05 were considered differentially represented.

### Microbial Function Prediction

Phylogenetic Investigation of Communities by Reconstruction of Unobserved States 2 (PICRUSt2; Langille et al., 2013; Douglas et al., 2020) was used to predict metagenome functions from 16S rRNA data, and the metagenomes were aligned to the Kyoto Encyclopedia of Genes and Genomes (KEGG) bioinformatics database. The Nearest Sequenced Taxon Index (NSTI) score, which measures the average distance between OTUs and their nearest sequenced genome representatives, was used as an indicator for the accuracy of PICRUSt (Langille et al., 2013). Significantly different KEGG pathways between seawater samples and intracolonial samples were identified and visualized by STAMP’s implementation (Parks et al., 2014) of a two-sided Welch’s *t* test with a Storey FDR adjusted q value <0.05. The final data were filtered to remove features with small effect sizes by considering only features with the ratio of the proportions were larger than 2 and the difference in proportions were larger than 0.5 (effect size<2.00).

## Results

### Sequencing and Quality Control

As the intracolonial fluid samples exacted from *P. globosa* colonies were mixture of microbiota and *P. globosa* cells (since individual *Phaeocystis* cells were likely released upon colony collapse as the internal fluid was removed), co-amplification of chloroplast DNA by 338F-806R was observed and resulted in 92.1% OTUs annotated as *P. globosa* chloroplast DNA in intracolonial fluid samples (Supplementary Figure S1). Therefore, primer pair 799F-1193R, which have been shown to perform better in plant-microbiota research (Beckers et al., 2016), were selected to remove chloroplast contamination. After being rarefied and filtered from the low abundance OTUs, none of OTUs were assigned to chloroplast sequences, suggested the OTUs represented bacterial DNA.

Illumina MiSeq PE300 platform was used for sequencing and produced sequences of 300 bases. A total of 108,764 high-quality 16S rRNA sequences were obtained from seawater samples (*n*=3) and intracolonial fluid samples (*n*=3). After clustering all sequences at a similarity threshold of 97%, being rarefied and filtered, 79,032 raw sequences assigned to 199 OTUs were established (Supplementary Table S1). Although only rarefaction curves of internal fluid samples reached an asymptote and rarefaction curves of seawater samples did not, the sequencing effort did detect the majority of species present in both samples. The result demonstrated that sequencing depth was sufficient to represent the total diversity of the microbial assemblage (Supplementary Figure S2).

### Microbial Diversity and Composition

Accumulation of chlorophyll along the coast was observed from satellite image (Figure 1), especially in our sampling area, where chlorophyll concentrations were greater than 9μgl^−1^. Based on long-term observation along Binh Thuan Province, upwelling occurred during the southwest monsoon in summer, and it caused blooms of diatom, *P. globosa* and dinoflagellate successively (Tang et al., 2004; Doan-Nhu et al., 2010; Smith et al., 2014). Our direct sampling and microscopic observation have suggested that enhancement of chlorophyll at Phan Thiet during our work has been largely contributed by colonial *P. globosa*, although other autotrophic species were present. Water temperature was *ca.* 28°C.

**Figure 2:**
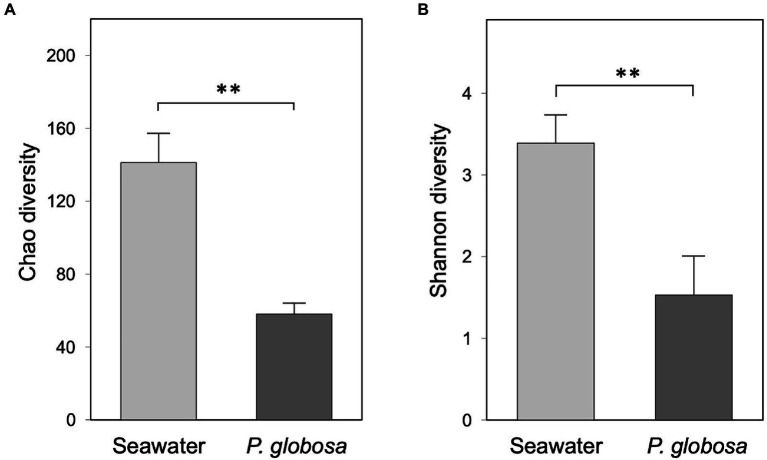
Bacterial community alpha-diversity of ambient seawater and *Phaeocystis globosa* intracolonial fluid as expressed by the Chao diversity **(A)** and Shannon diversity **(B)** indices. Values are means of triplicates ± SD. The asterisks on the bar chars represent the statistical significance of the difference (Student’s *t* test, ^**^ *p*<0.01).

The Chao and Shannon diversity indices of microbial assemblage were significantly higher in seawater than in intracolonial fluid (Student’s *t* test; Chao 1, *p*<0.01; Shannon: *p*<0.01; Figure 2), which indicates that ambient seawater possessed the higher levels of microbial richness and diversity, and conversely that the microbial diversity within *P. globosa* microenvironments was significantly reduced. This suggests that the microenvironment of the internal fluid in *P. globosa* provided a substantially different growth medium than that external to colonies.

**Figure 3:**
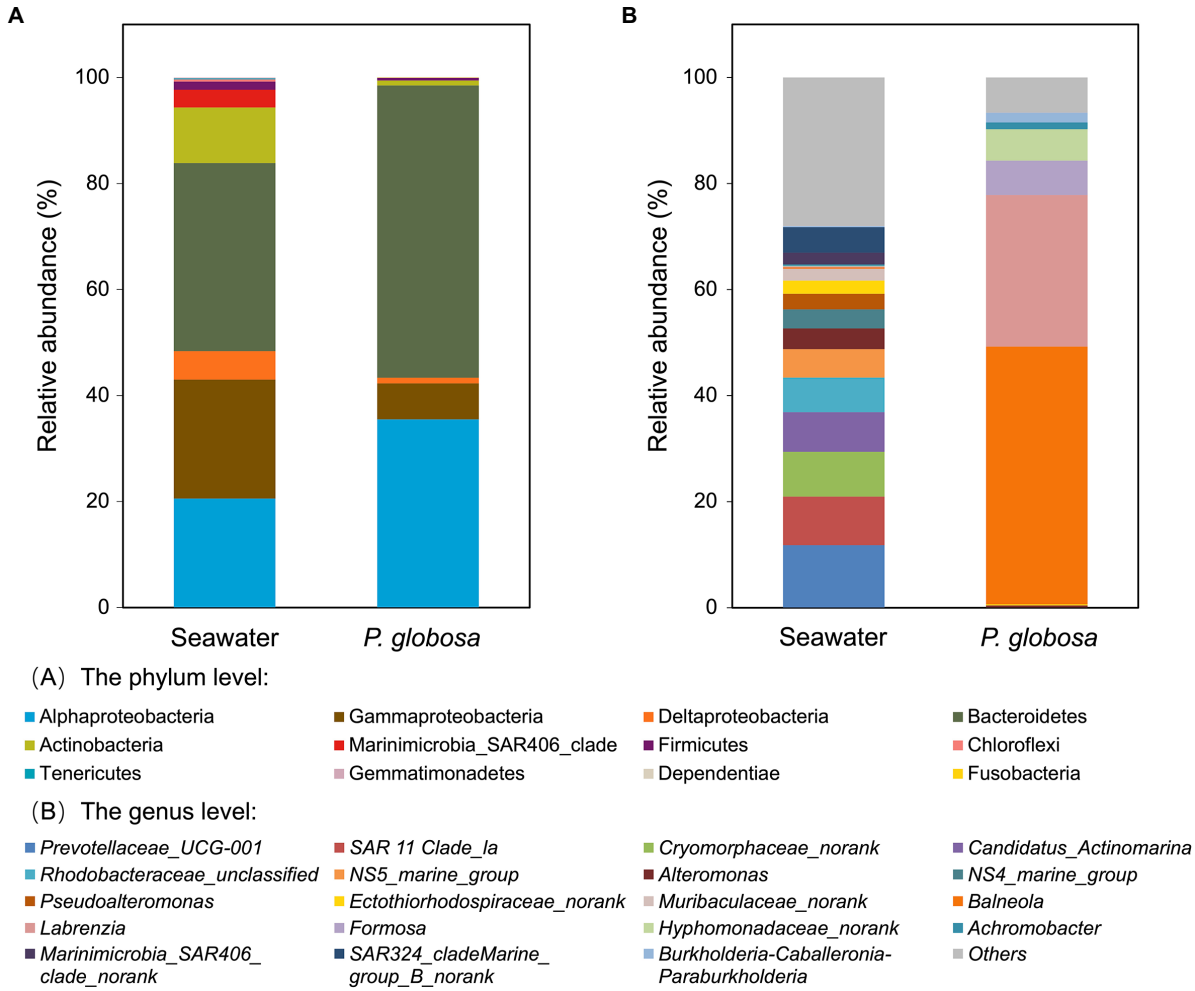
Bacterial composition of ambient seawater and *P. globosa* intracolonial fluid. The stacked bar plots show the relative abundance of operational taxonomic units (OTUs) at phylum levels **(A)** and genus levels **(B)**. Proteobacteria OTUs has been displayed at the subclass levels (alpha, beta, gamma and delta) (A). Each bar represents the average of pooled replicates.

At the phylum level, seawater samples were dominated by Proteobacteria (48.4%), Bacteroidetes (35.5%), and Actinobacteria (10.5%), which together contributed 94.3% of seawater microbiota, while Bacteroidetes (55.1%) and Proteobacteria (43.4%) composed 98.5% of the intracolonial fluid microbiota (Figure 3A, Supplementary Table S1). However, at the class level, great difference of Proteobacteria abundance was observed between seawater and intracolonial fluids microbiota, e.g., abundance of Alphaproteobacteria were 20.6 and 35.5%, respectively (Figure 3A). Other phyla, including Marinimicrobia, Firmicutes, shared the remaining portion, which was 5.7 and 1.5% of seawater and intracolonial fluid microbiota, respectively.

At the genus level, the dominant taxa in intracolonial fluids were distinct from those in seawater (Figure 3B). In intracolonial fluid, the four dominant genera (*Balneola,* 48.6%; *Labrezia,* 28.5%; *Formosa,* 6.5%; and *Hyphomonadaceae* unclassified, 5.9%) represented nearly 90% of the microbiota. In contrast, they were in extremely uncommon in seawater (0.3, 0.1,<0.1 and<0.1%, respectively). Moreover, none of the top five dominant bacterial genera in seawater, *Prevotellaceae*_UCG-001, SAR 11 Clade_la, *Cryomorphaceae*, *Candidatus_Actinomarina,* and *Rhodobacteraceae* were present in the intracolonial fluid microbiota (Figure 3B).

The results of the LEfSe identified the taxa enriched in intracolonial fluid of *P. globosa* colonies. The 50 most abundant OTUs comprised 90.8% of all microbiota, and they were used for enriched OTUs analysis. Seven OTUs were significantly enriched in intracolonial fluid, including the four dominant genera *Balneola*, *Labrezia*, *Formosa,* and *Hyphomonadaceae* unclassified (Supplementary Figure S3).

### Predicted Microbial Functioning

**Figure 4:**
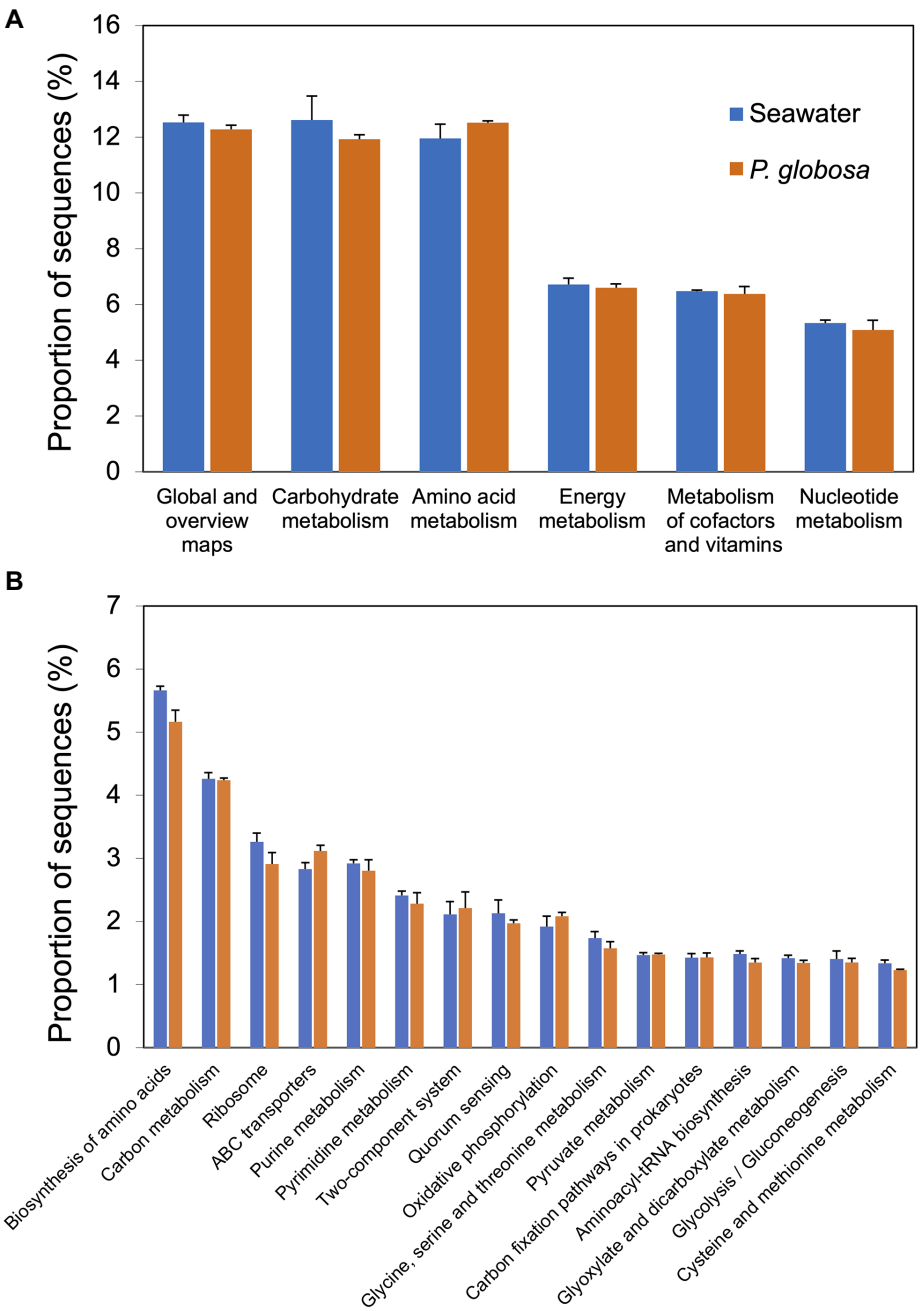
Predicted microbial functions of samples collected from ambient seawater and *P. globosa* intracolonial fluid. Functions were categorized to KEGG pathway level 2 and level 3 using PICRUSt software. The top six dominant functions categorized to level 2 **(A)** and the top fifteen dominant functions categorized to level 3 **(B)** were ordered from largest contribution (left) to smallest contribution (right).

**Figure 5:**
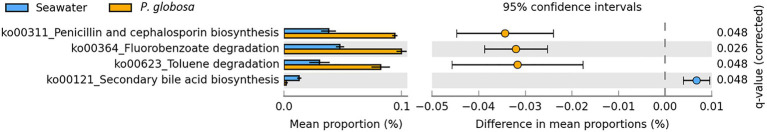
Predicted microbial functions with significantly difference in samples collected from ambient seawater and *P. globosa* intracolonial fluid. Differential abundance between samples was determined using a two-sided Welch’s *t* test, with a Storey FDR, adjusted q value <0.05 and followed by an effect size filter (ratio of proportions effect size <2.00).

Functional prediction was conducted using PICRUSt2, a software tool that predicts the functional profile of a microbial consortia based on 16S rRNA sequences (Langille et al., 2013). Prediction accuracy was first evaluated by NSTI scores. Mean (and standard deviation) NSTI scores of seawater samples and intracolonial fluid samples were 0.258 (0.031) and 0.111 (0.002), respectively (Supplementary Table S2). A total of 5746 KEGG orthology (KO) categories were obtained. PICRUSt2 analysis predictions at KEGG level 2 revealed that all of the six dominant KEGG categories belonged to metabolism category at KEGG level 1, including global and overview maps, carbohydrate metabolism, amino acid metabolism, energy metabolism, metabolism of cofactors and vitamins, and nucleotide metabolism (Figure 4A). These functional categories were relatively consistent in both sample types and comprised 55.6% (seawater) and 54.8% (intracolonial fluid) of the predicted level 2 functional categories, respectively (Figure 4A; Supplementary Table S3). According to the KEGG hierarchical level 3 classification, 303 metabolic pathways were predicted (Supplementary Table S3). Seawater and intracolonial fluid microbiota shared the same top 15 KEGG pathways, which contributed 36.4 and 35.3%, respectively (Figure 4B). The dominant functions were biosynthesis of amino acids (ko01230) and carbon metabolism (ko01200), contributing 5.7 and 4.3% in seawater, and 5.2 and 4.2% in intracolonial fluid, respectively (Figure 4B; Supplementary Table S3). Four pathways were identified as being significantly different between seawater and intracolonial fluid samples (Figure 5). Secondary bile acid biosynthesis (ko00121) was the only pathway enriched in seawater microbiota, while KEGG pathways involved in penicillin and cephalosporin biosynthesis (ko00311), fluorobenzoate degradation (ko00364), and toluene degradation (ko00623) were enriched in intracolonial fluid.

## Discussion

DNA sequencing of 16S rRNA gene fragments revealed that the microbial assemblage in the intracolonial fluid of *P. globosa* colonies is significantly different from that in ambient seawater. As the intracolonial fluid extracted from *P. globosa* colonies contained a mixture of bacteria and *P. globosa* cells, we used a specific 799F-1193R primer set to minimize contamination of chloroplast sequences (Chelius and Triplett, 2001; Bodenhausen et al., 2013), which has been used in several studies on bacteria associated with plants and macroalgae (Bulgarelli et al., 2012; Vieira et al., 2016; Serebryakova et al., 2018). This primer set displayed very low amplification of non-target DNA and retrieved high number of OTUs as well as exhibiting high Inverse Simpson diversity (Beckers et al., 2016). The use of 799F-1193R primer pair also revealed that Shannon diversity of bacteria from seawater was high and comparable to previous studies (Wang et al., 2019). Although PCR amplification bias introduced by primer selection was inevitable, the primer pair 799F-1193R was well suited for *Phaeocystis*-associated microbiome analysis.

Our results indicated that both the microbial richness and diversity of the surrounding water were significantly higher than those of the intracolonial fluid, suggesting that the microenvironment of colonies allowed the development of an independent consortia from that outside the colony. Moreover, the four dominant genera, which represented 89.6% of the intracolonial microbiota, were present in extremely low abundances in seawater. This is consistent with previous research that reported that the diatom *Thalassiosira rotula*-associated microbial assemblages have lowered diversity and are generally dominated by fewer bacterial species (Mönnich et al., 2020). Phytoplankton microenvironments may be much more common in the environment than previously thought and have considerable impacts on biogeochemical cycles.

Although numerous studies revealed the mutually beneficial relationship between phytoplankton and their associated bacteria, there is currently no unifying theory for the mechanism of bacterial assembly on marine phytoplankton. Two hypotheses have been proposed to explain the development of specific phytoplankton-associated bacterial consortia. One is the lottery hypothesis, which implies whoever “gets there first” ultimately dominates the consortia (Munday, 2004). Another hypothesis is “habitat filtering effect,” which emphasizes interactions and the selection from the host (Costello et al., 2012). The second theory has been supported by numerous direct experimental investigations, including the recent replicated and reproducible primary colonization experiment (Mönnich et al., 2020). *Phaeocystis* colonies likely form from solitary cells that multiply and remain together, ultimately forming colonies (Mathot et al., 2000), although it is also possible for colonies to fragment and create smaller colonies. Recently a colony formation model of *P. globosa* has been proposed, which suggested that even a single solitary cell with flagella can form the polysaccharide matrix (Zhang et al., 2020). Thus, the volume of seawater inside the matrix would initially be very small and contain only a limited number of bacteria, implying that either a few specific *P. globosa*-associated bacteria or stochastic bacteria in the ambient seawater would have been initially enclosed in the envelope. However, as colonies grew larger, the microenvironment becomes altered, being substantially different from ambient seawater [i.e., it has greater DOC concentrations (Smith et al., 2014) and elevated pH levels (Lubbers et al., 1990)], allowing for a selection of bacterial forms as the colony grows and continues to alter the intracolonial fluid.

Many observations confirm that phytoplankton-associated bacteria are often restricted to only a small number of groups, dominated by specific members of Bacteroidetes, Alphaproteobacteria, and Gammaproteobacteria (Buchan et al., 2014). The most enriched OTUs in the intracolonial fluid were identified as belonging to the genera *Balneola* and *Labrezia*, which are Bacteroidetes and Alphaproteobacteria, respectively. The most dominant OTU (*Balneola*) comprised 48.6% of the intracolonial fluid sequences. *Balneola* has been frequently observed to associate with marine phytoplankton, including *Emiliania huxley* and *Cochlodinium polykrikoides* (Taniguchi et al., 2011; Rosana et al., 2016). An arabinogalactan utilization gene cluster present in *Balneola* sp. genome indicated its ability to degrade polysaccharide, thus the heterotrophic bacteria could use polysaccharide synthesized by *P. globosa* as its carbon source. Moreover, *Balneola* has been implicated in the degradation of aromatic benzenoid compounds such as toluene (Li et al., 2012). Aromatic benzenoid compounds can be significant organic contaminants, but some benzenoid compounds serve as a biochemical defense when plants or microorganisms encounter stress (Misztal et al., 2015). No evidence is available for toluene production by *P. globosa*, although significant toluene fluxes to the atmosphere from *Emiliania huxleyi* blooms (a haptophyte like *P. globosa*), have been observed (Misztal et al., 2015). Isotopic labeling experiments have confirmed that plants release toluene and increase toluene production under stress conditions (Heiden et al., 1999; Misztal et al., 2015). Although the biological function of toluene in plants and phytoplankton remains unclear, we speculate that toluene accumulated in *P. globosa* colonies could provide carbon source to *Balneola*.

The second dominant OTU was classified as *Labrenzia*, which frequently forms close associations with a variety of marine autotrophs including diatoms, dinoflagellates, green and red algae (Weber and King, 2007). However, the close relationship between *Labrenzia* and *P. globosa*, and the biological function of enriched *Labrenzia* in *P. globosa* intracolonial fluid, have not been reported. It has long been recognized that *P. globosa* is an important DSMP producer and consumer (Keller et al., 1989; Stefels and Van Boekel, 1993). However, the role of the marine heterotrophic bacteria *Labrenzia* in DMSP producing and consuming has been demonstrated recently (Curson et al., 2017). In addition to serving as an osmolyte in many marine algae, including prymnesiophytes and dinoflagellates, DMSP and DMS also function as antioxidants under stressful conditions (e.g., high solar photon fluxes and nutrient limitation), which result in substantial increases in cellular DMSP and DMS concentrations (Sunda et al., 2002; Brisbin and Mitarai, 2019). Marine phytoplankton will encounter challenges caused by global change in the future, and as a result, cellular DMSP and DMS production may increase due to this stress. If this were to occur, release of these volatile sulfur compounds to the atmosphere will increase, enhancing atmospheric feedback loops that influence global climate and hydrological cycles. Research to date has not focused on the source of DMSP and DMS in *P. globosa* colonies, but future research should determine the dominant source of DMS/DMSP and consider the interaction between the two biotic components.

In the present study, dominant bacteria in the intracolonial fluid were completely different from the dominant bacteria observed by Xu et al. (2021) during *P. globosa* blooms, neither in free-living fraction (0.22–3μm) nor in particle-attached fraction (3–20μm). However, *NS5* marine group, which belongs to Proteobacteria, was observed as one of the dominant bacteria in seawater free-living fractions at the bloom site from both studies. The results suggested the significance of microenvironment enclosed by colonial envelope, which established the distinct bacterial community. The interaction between heterotrophic bacteria and *P. globosa* solitary cells in the microenvironment might benefit the development of *P. globosa* colony.

Bioinformatic prediction by PICRUSt offers an effective way to understand the function of bacteria inside *P. globosa* colonies when metagenomic data are not available. However, methods, including NSTI scores, should be used to test the reliability of PICRUSt. Generally, accuracy of PICRUSt decreased with increasing NSTI score, but still produced reliable results for a dataset of soil samples with a mean NSTI score of 0.17 (Langille et al., 2013). In the present study, mean NSTI scores were relatively low (0.111) for intracolonial fluid samples, and relatively high for seawater samples (0.258). Langille et al. (2013) indicated that environments containing much unexplored diversity, such as the Guerrero Negro hypersaline microbial mats, showed a markedly lower accuracy with a mean NSTI score of 0.23. The relatively high NSTI scores obtained here indicate that the PICRUSt predictions must be treated with caution. The results, however, still provide some interesting insights into potential bacterial community functioning, that should be confirmed in future with more reliable and quantitative studies.

PICRUSt prediction revealed that the 15 abundant KEGG level 2 pathways were the same in *P. globosa* intracolonial fluid and ambient seawater, which demonstrated that both microbiomes have the same basic biological functions. However, significant functional differences were observed between two microbiomes by STAMP analysis. Surprisingly, the KEGG pathway of penicillin and cephalosporin biosynthesis (ko00311) was significantly enriched in intracolonial bacteria communities. Synthesis of antibacterial compounds is one mechanism by which one particular species of bacteria can out-compete another and increase the successful colonization associated with phytoplankton. The widespread marine roseobacter *Phaeobacter inhibens* produces the broad-spectrum antibiotic tropodithietic acid (TDA), which confers to it a competitive advantage (Majzoub et al., 2019). Thus, *P. inhibens* dominates the diatom *T. rotula* associated microbiota (Majzoub et al., 2019); it also colonizes the surface of *Ulva australis* (Rao et al., 2006). Our results suggest that elevated bacterial competition in *P. globosa* intracolonial fluid *via* the release of antibacterial compounds likely resulted in lower species diversity of the *P. globosa*-associated microbiota than that in the ambient seawater. Consistent with the function of dominant genus *Balneola* in the intracolonial fluid, the aromatic compound degradation pathways were also depicted by PICRUSt prediction. Although the source of the aromatic compounds in the intracolonial fluid is unknown, the predicted function suggests that the associated bacteria could use fluorobenzoate and toluene as a carbon and energy source.

Based on the dominated OTUs and the enriched predicted KEGG pathways, our study suggests that the *P. globosa* colonial envelope acts as a selective mechanism, and allows the development of a bacterial consortia and their specific functions. Furthermore, upregulated synthesis of antibacterial compounds also promoted the specificity of intracolonial bacteria. Enriched bacteria in the intracolonial fluid involved in DMSP and DMS production, which may affect DMSP and DMS synthesis by *P. globosa*, thus have a substantial influence on the sulfate cycle of the ocean. The results presented here highlight the close interaction between *P. globosa* and bacteria in the intracolonial fluid. However, the specific mechanisms are still unknown. For example, although both the toluene-degrader *Balneola* and the predicted function of toluene degradation have been suggested, the concentration of toluene inside the colonies is unknown. Thus, further research is needed to determine whether *P. globosa* can produce toluene, under what conditions this production is enhanced, and to investigate the interaction between *Balneola* and *P. globosa*. In addition, the contribution of *Balneola* and *Labrezia* in *P. globosa* intracolonial fluids should be confirmed by qPCR. While metabolic function analysis was only predicted by PICRUSt, its accuracy relies on the availability of completely sequenced genomes for the representative organisms (Wilkinson et al., 2018). Therefore, metagenomics and metatranscriptomics analysis are needed to verify the actual bacterial structure and function of *P. globosa*-associated communities to reveal the interaction between microbiota and *P. globosa*.

## Conclusion

We assessed the bacterial composition and their predicted functions in *P. globosa* intracolonial fluids. The results indicated that bacterial richness and diversity were significantly lower in intracolonial fluid than that in ambient seawater due to the selection by unique intracolonial microenvironment. In addition, the close interaction between *P. globosa* solitary cells and heterotrophic bacteria might benefit the development of *P. globosa* and further impact the global biogeochemical cycles. In summary, the results suggests that the *P. globosa* colonial envelope acts as a selective mechanism and allows the development of a bacterial consortia and their specific functions.

## Data Availability Statement

The raw reads are available by the valid accession number: PRJNA720571.

## Author Contributions

ZZ, RM, WS, HD-N, and LN-N designed the experiment. ZZ, RM, WS, and HD-N collected sample in Viet Nam. 16S rDNA sequence analyses and bioinformatics analyses were conducted by ZZ and XJ. ZZ wrote the manuscript with contributions from RM and XJ. WS and HD-N revised the manuscript. All authors agree to be listed in this manuscript and approve the submitted version of the manuscript.

## Funding

This work was supported by grants from the National Foundation for Science and Technology Development (NAFOSTED) of Viet Nam awarded to HD-N and LN-N (DFG 106-NN.06-2016.78).

## Conflict of Interest

The authors declare that the research was conducted in the absence of any commercial or financial relationships that could be construed as a potential conflict of interest.

## Publisher’s Note

All claims expressed in this article are solely those of the authors and do not necessarily represent those of their affiliated organizations, or those of the publisher, the editors and the reviewers. Any product that may be evaluated in this article, or claim that may be made by its manufacturer, is not guaranteed or endorsed by the publisher.

## References

[ref1] Baumann M. E. M. Lancelot C. Brandini F. P. Sakshaug E. John D. M. (1994). The taxonomic identity of the cosmopolitan prymnesiophyte *Phaeocystis*: a morphological and ecophysiological approach. J. Mar. Syst. 5, 5–22. doi: 10.1016/0924-7963(94)90013-2

[ref2] Beckers B. De Beeck M. O. Thijs S. Truyens S. Weyens N. Boerjan W.. (2016). Performance of 16s rDNA primer pairs in the study of rhizosphere and endosphere bacterial microbiomes in metabarcoding studies. Front. Microbiol. 7:650. doi: 10.3389/fmicb.2016.00650, PMID: 27242686 PMC4865482

[ref3] Bodenhausen N. Horton M. W. Bergelson J. (2013). Bacterial communities associated with the leaves and the roots of *Arabidopsis thaliana*. PLoS One 8:e56329. doi: 10.1371/journal.pone.0056329, PMID: 23457551 PMC3574144

[ref4] Brisbin M. M. Mitarai S. (2019). Differential gene expression supports a resource-intensive, defensive role for Colony production in the bloom-forming haptophyte, *Phaeocystis globosa*. J. Eukaryot. Microbiol. 66, 788–801. doi: 10.1111/jeu.12727, PMID: 30860641 PMC6766888

[ref5] Buchan A. Lecleir G. R. Gulvik C. A. Gonzalez J. M. (2014). Master recyclers: features and functions of bacteria associated with phytoplankton blooms. Nat. Rev. Microbiol. 12, 686–698. doi: 10.1038/nrmicro3326, PMID: 25134618

[ref6] Bulgarelli D. Rott M. Schlaeppi K. Van Themaat E. V. L. Ahmadinejad N. Assenza F.. (2012). Revealing structure and assembly cues for *Arabidopsis* root-inhabiting bacterial microbiota. Nature 488, 91–95. doi: 10.1038/nature11336, PMID: 22859207

[ref7] Cadee G. C. Hegeman J. (2002). Phytoplankton in the Marsdiep at the end of the 20th century; 30 years monitoring biomass, primary production, and *Phaeocystis* blooms. J. Sea Res. 48, 97–110. doi: 10.1016/S1385-1101(02)00161-2

[ref8] Chelius M. K. Triplett E. W. (2001). The diversity of archaea and bacteria in association with the roots of *Zea mays* L. Microb. Ecol. 41, 252–263. doi: 10.1007/s002480000087, PMID: 11391463

[ref9] Chen S. Zhou Y. Chen Y. Gu J. (2018). fastp: an ultra-fast all-in-one FASTQ preprocessor. Bioinformatics 34, 884–890. doi: 10.1093/bioinformatics/bty560 30423086 PMC6129281

[ref10] Costello E. K. Stagaman K. Dethlefsen L. Bohannan B. J. M. Relman D. A. (2012). The application of ecological theory toward an understanding of the human microbiome. Science 336, 1255–1262. doi: 10.1126/science.1224203, PMID: 22674335 PMC4208626

[ref11] Curson A. R. J. Liu J. Martinez A. B. Green R. T. Chan Y. Carrion O.. (2017). Dimethylsulfoniopropionate biosynthesis in marine bacteria and identification of the key gene in this process. Nat. Microbiol. 2:17009. doi: 10.1038/nmicrobiol.2017.9, PMID: 28191900

[ref12] Delmont T. O. Eren A. M. Vineis J. H. Post A. F. (2015). Genome reconstructions indicate the partitioning of ecological functions inside a phytoplankton bloom in the Amundsen Sea. Antarctica. Front. Microbiol. 6:1090. doi: 10.3389/fmicb.2015.01090, PMID: 26579075 PMC4620155

[ref13] Delmont T. O. Hammar K. M. Ducklow H. W. Yager P. L. Post A. F. (2014). *Phaeocystis antarctica* blooms strongly influence bacterial community structures in the Amundsen Sea polynya. Front. Microbiol. 5:646. doi: 10.3389/fmicb.2014.00646, PMID: 25566197 PMC4271704

[ref14] Doan-Nhu H. Nguyen-Ngoc L. Dippner J. W. (2010). Development of *Phaeocystis globosa* blooms in the upwelling waters of the south central coast of Viet Nam. J. Mar. Syst. 83, 253–261. doi: 10.1016/j.jmarsys.2010.04.015

[ref15] Douglas G. M. Maffei V. J. Zaneveld J. R. Yurgel S. N. Brown J. R. Taylor C. M.. (2020). PICRUSt2 for prediction of metagenome functions. Nat. Biotechnol. 38, 685–688. doi: 10.1038/s41587-020-0548-6, PMID: 32483366 PMC7365738

[ref16] Edgar R. C. (2013). UPARSE: highly accurate OTU sequences from microbial amplicon reads. Nat. Methods 10, 996–998. doi: 10.1038/nmeth.2604, PMID: 23955772

[ref17] Gradoville M. R. Crump B. C. Letelier R. M. Church M. J. White A. E. (2017). Microbiome of *Trichodesmium* colonies from the North Pacific subtropical gyre. Front. Microbiol. 8:1122. doi: 10.3389/fmicb.2017.01122 28729854 PMC5498550

[ref18] Hamm C. E. (2000). Architecture, ecology and biogeochemistry of *Phaeocystis* colonies. J. Sea Res. 43, 307–315. doi: 10.1016/S1385-1101(00)00014-9

[ref19] Hamm C. E. Simson D. A. Merkel R. Smetacek V. (1999). Colonies of *Phaeocystis globosa* are protected by a thin but tough skin. Mar. Ecol. Prog. Ser. 187, 101–111. doi: 10.3354/meps187101

[ref20] Heiden A. C. Kobel K. Komenda M. Koppmann R. Shao M. Wildt J. (1999). Toluene emissions from plants. Geophys. Res. Lett. 26, 1283–1286. doi: 10.1029/1999GL900220

[ref21] Jakobsen H. H. Tang K. W. (2002). Effects of protozoan grazing on colony formation in *Phaeocystis globosa* (Prymnesiophyceae) and the potential costs and benefits. Aquat. Microb. Ecol. 27, 261–273. doi: 10.3354/ame027261

[ref22] Keller M. D. Bellows W. K. Guillard R. R. L. (1989). “Dimethyl sulfide production in marine phytoplankton” in Biogenic Sulfur in the Environment. eds. Saltzman E. S. Cooper W. J. (Washington DC: American Chemical Society), 167–182.

[ref23] Klawonn I. Eichner M. J. Wilson S. T. Moradi N. Thamdrup B. Kuemmel S.. (2020). Distinct nitrogen cycling and steep chemical gradients in *Trichodesmium* colonies. ISME J. 14, 399–412. doi: 10.1038/s41396-019-0514-9, PMID: 31636364 PMC6976679

[ref24] Langille M. G. I. Zaneveld J. Caporaso J. G. Mcdonald D. Knights D. Reyes J. A.. (2013). Predictive functional profiling of microbial communities using 16S rRNA marker gene sequences. Nat. Biotechnol. 31, 814–821. doi: 10.1038/nbt.2676, PMID: 23975157 PMC3819121

[ref25] Li H. Zhang Q. Wang X.-L. Ma X.-Y. Lin K.-F. Liu Y.-D.. (2012). Biodegradation of benzene homologues in contaminated sediment of the East China Sea. Bioresour. Technol. 124, 129–136. doi: 10.1016/j.biortech.2012.08.033 22989641

[ref26] Lubbers G. Gieskes W. Castilho P. Del Salomons W. Bril J. (1990). Manganese accumulation in the high pH microenvironment of *Phaeocystis* sp. (Haptophyceae) colonies from the North Sea. Mar. Ecol. Prog. Ser. 59, 285–293. doi: 10.3354/meps059285

[ref27] Madhupratap M. Sawant S. Gauns M. (2000). A first report on a bloom of the marine prymnesiophycean *Phaeocystis globosa* from the Arabian Sea. Oceanol. Acta 23, 83–90. doi: 10.1016/s0399-1784(00)00109-2

[ref28] Magoc T. Salzberg S. L. (2011). FLASH: fast length adjustment of short reads to improve genome assemblies. Bioinformatics 27, 2957–2963. doi: 10.1093/bioinformatics/btr507, PMID: 21903629 PMC3198573

[ref29] Majzoub M. E. Beyersmann P. G. Simon M. Thomas T. Brinkhoff T. Egan S. (2019). *Phaeobacter inhibens* controls bacterial community assembly on a marine diatom. FEMS Microbiol. Ecol. 95:fiz060. doi: 10.1093/femsec/fiz060, PMID: 31034047

[ref30] Mathot S. Smith W. O. Carlson C. A. Garrison D. L. Gowing M. M. Vickers C. L. (2000). Carbon partitioning within *Phaeocystis antarctica* (Prymnesiophyceae) colonies in the Ross Sea. Antarctica. J. Phycol. 36, 1049–1056. doi: 10.1046/j.1529-8817.2000.99078.x

[ref31] Misztal P. K. Hewitt C. N. Wildt J. Blande J. D. Eller A. S. D. Fares S.. (2015). Atmospheric benzenoid emissions from plants rival those from fossil fuels. Sci. Rep. 5:12064. doi: 10.1038/srep12064 26165168 PMC4499884

[ref32] Mönnich J. Tebben J. Bergemann J. Case R. Wohlrab S. Harder T. (2020). Niche-based assembly of bacterial consortia on the diatom *Thalassiosira rotula* is stable and reproducible. ISME J. 14, 1614–1625. doi: 10.1038/s41396-020-0631-5, PMID: 32203123 PMC7242391

[ref33] Munday P. L. (2004). Competitive coexistence of coral-dwelling fishes: The lottery hypothesis revisited. Ecology 85, 623–628. doi: 10.1890/03-3100

[ref34] Parks D. H. Tyson G. W. Hugenholtz P. Beiko R. G. (2014). STAMP: statistical analysis of taxonomic and functional profiles. Bioinformatics 30, 3123–3124. doi: 10.1093/bioinformatics/btu494, PMID: 25061070 PMC4609014

[ref35] Ploug H. Stolte W. Jorgensen B. B. (1999). Diffusive boundary layers of the colony-forming plankton alga *Phaeocystis* sp - implications for nutrient uptake and cellular growth. Limnol. Oceanogr. 44, 1959–1967. doi: 10.4319/lo.1999.44.8.1959

[ref36] Qi Y. Z. Chen J. F. Wang Z. H. Xu N. Wang Y. Shen P. P.. (2004). Some observations on harmful algal bloom (HAB) events along the coast of Guangdong, southern China in 1998. Hydrobiologia 512, 209–214. doi: 10.1023/B:HYDR.0000020329.06666.8c

[ref37] Rao D. Webb J. S. Kjelleberg S. (2006). Microbial colonization and competition on the marine alga *Ulva australis*. Appl. Environ. Microbiol. 72, 5547–5555. doi: 10.1128/AEM.00449-06, PMID: 16885308 PMC1538698

[ref38] Rosana A. R. R. Orate F. D. Xu Y. Simkus D. N. Bramucci A. R. Boucher Y.. (2016). Draft genome sequences of seven bacterial strains isolated from a polymicrobial culture of coccolith-bearing (C-type) *Emiliania huxleyi* M217. Genome Announc. 4:0067316. doi: 10.1128/genomeA.00673-16 PMC4945805 27417845

[ref39] Rousseau V. Vaulot D. Casotti R. Cariou V. Lenz J. Gunkel J.. (1994). The life cycle of *Phaeocystis* (Prymnesiophycaea): evidence and hypotheses. J. Mar. Syst. 5, 23–39. doi: 10.1016/0924-7963(94)90014-0

[ref40] Schoemann V. Becquevort S. Stefels J. Rousseau W. Lancelot C. (2005). *Phaeocystis* blooms in the global ocean and their controlling mechanisms: a review. J. Sea Res. 53, 43–66. doi: 10.1016/j.seares.2004.01.008

[ref41] Segata N. Izard J. Waldron L. Gevers D. Miropolsky L. Garrett W. S.. (2011). Metagenomic biomarker discovery and explanation. Genome Biol. 12:R60. doi: 10.1186/gb-2011-12-6-r60, PMID: 21702898 PMC3218848

[ref42] Serebryakova A. Aires T. Viard F. Serrao E. A. Engelen A. H. (2018). Summer shifts of bacterial communities associated with the invasive brown seaweed *Sargassum muticum* are location and tissue dependent. PLoS One 13:e0206734. doi: 10.1371/journal.pone.0206734, PMID: 30517113 PMC6281184

[ref43] Seymour J. R. Amin S. A. Raina J.-B. Stocker R. (2017). Zooming in on the phycosphere: the ecological interface for phytoplankton-bacteria relationships. Nat. Microbiol. 2:17065. doi: 10.1038/nmicrobiol.2017.65 28555622

[ref44] Smith W. O. Jr. Liu X. Tang K. W. Delizo L. M. Nhu Hai D. Ngoc Lam N.. (2014). Giantism and its role in the harmful algal bloom species *Phaeocystis globosa*. Deep-Sea ResPt II 101, 95–106. doi: 10.1016/j.dsr2.2012.12.005

[ref45] Stefels J. Van Boekel W. H. M. (1993). Production of DMS from dissolved DMSP in axenic cultures of the marine phytoplankton species *Phaeocystis* sp. Mar. Ecol. Prog. Ser. 97, 11–18. doi: 10.3354/meps097011

[ref46] Sunda W. Kieber D. J. Kiene R. P. Huntsman S. (2002). An antioxidant function for DMSP and DMS in marine algae. Nature 418, 317–320. doi: 10.1038/nature00851, PMID: 12124622

[ref47] Tang D. L. Kawamura H. Doan-Nhu H. Takahashi W. (2004). Remote sensing oceanography of a harmful algal bloom off the coast of southeastern Vietnam. J. Geophys. Res. Oceans 109:C03014. doi: 10.1029/2003JC002045

[ref48] Taniguchi A. Tada Y. Hamasaki K. (2011). Seasonal variations in the community structure of actively growing bacteria in neritic waters of Hiroshima Bay, western Japan. Microbes Environ. 26, 339–346. doi: 10.1264/jsme2.ME11212, PMID: 21791885

[ref49] Vanrijssel M. Hamm C. E. Gieskes W. W. C. (1997). *Phaeocystis globosa* (Prymnesiophyceae) colonies: hollow structures built with small amounts of polysaccharides. Eur. J. Phycol. 32, 185–192.

[ref50] Vieira C. Engelen A. H. Guentas L. Aires T. Houlbreque F. Gaubert J.. (2016). Species specificity of bacteria associated to the brown seaweeds *Lobophora* (Dictyotales, Phaeophyceae) and their potential for induction of rapid coral bleaching in *Acropora muricata*. Front. Microbiol. 7:316. doi: 10.3389/fmicb.2016.00316, PMID: 27047453 PMC4800410

[ref51] Wang Q. Garrity G. M. Tiedje J. M. Cole J. R. (2007). Naive Bayesian classifier for rapid assignment of rRNA sequences into the new bacterial taxonomy. Appl. Environ. Microbiol. 73, 5261–5267. doi: 10.1128/AEM.00062-07, PMID: 17586664 PMC1950982

[ref52] Wang Z. Juarez D. L. Pan J.-F. Blinebry S. K. Gronniger J. Clark J. S.. (2019). Microbial communities across nearshore to offshore coastal transects are primarily shaped by distance and temperature. Environ. Microbiol. 21, 3862–3872. doi: 10.1111/1462-2920.14734, PMID: 31286605

[ref53] Weber C. F. King G. M. (2007). Physiological, ecological, and phylogenetic characterization of Stappia, a marine CO-oxidizing bacterial genus. Appl. Environ. Microbiol. 73, 1266–1276. doi: 10.1128/AEM.01724-06, PMID: 17142374 PMC1828652

[ref54] Wilkinson T. J. Huws S. A. Edwards J. E. Kingston-Smith A. H. Siu-Ting K. Hughes M.. (2018). CowPI: A rumen microbiome Focussed version of the PICRUSt functional inference software. Front. Microbiol. 9:1095. doi: 10.3389/fmicb.2018.01095 29887853 PMC5981159

[ref55] Xu S. He C. Song S. Li C. (2021). Spatiotemporal dynamics of marine microbial communities following a *Phaeocystis* bloom: biogeography and co-occurrence patterns. Environ. Microbiol. Rep. 13, 294–308. doi: 10.1111/1758-2229.12929, PMID: 33527743

[ref56] Zhang S.-F. Zhang K. Cheng H.-M. Lin L. Wang D.-Z. (2020). Comparative transcriptomics reveals colony formation mechanism of a harmful algal bloom species *Phaeocystis globosa*. Sci. Total Environ. 719:137454. doi: 10.1016/j.scitotenv.2020.137454 32114233

