# Supplementary material for: Bacterial Composition Associated With Giant Colonies of the Harmful Algal Species Phaeocystis globosa
Source: Front Microbiol. 2021 Sep 17;12:737484. doi: 10.3389/fmicb.2021.737484 (PMC8555426; doi:10.3389/fmicb.2021.737484)
Supplement: Supplementary file 4 [file Data_Sheet_4.docx]

Supplementary Material


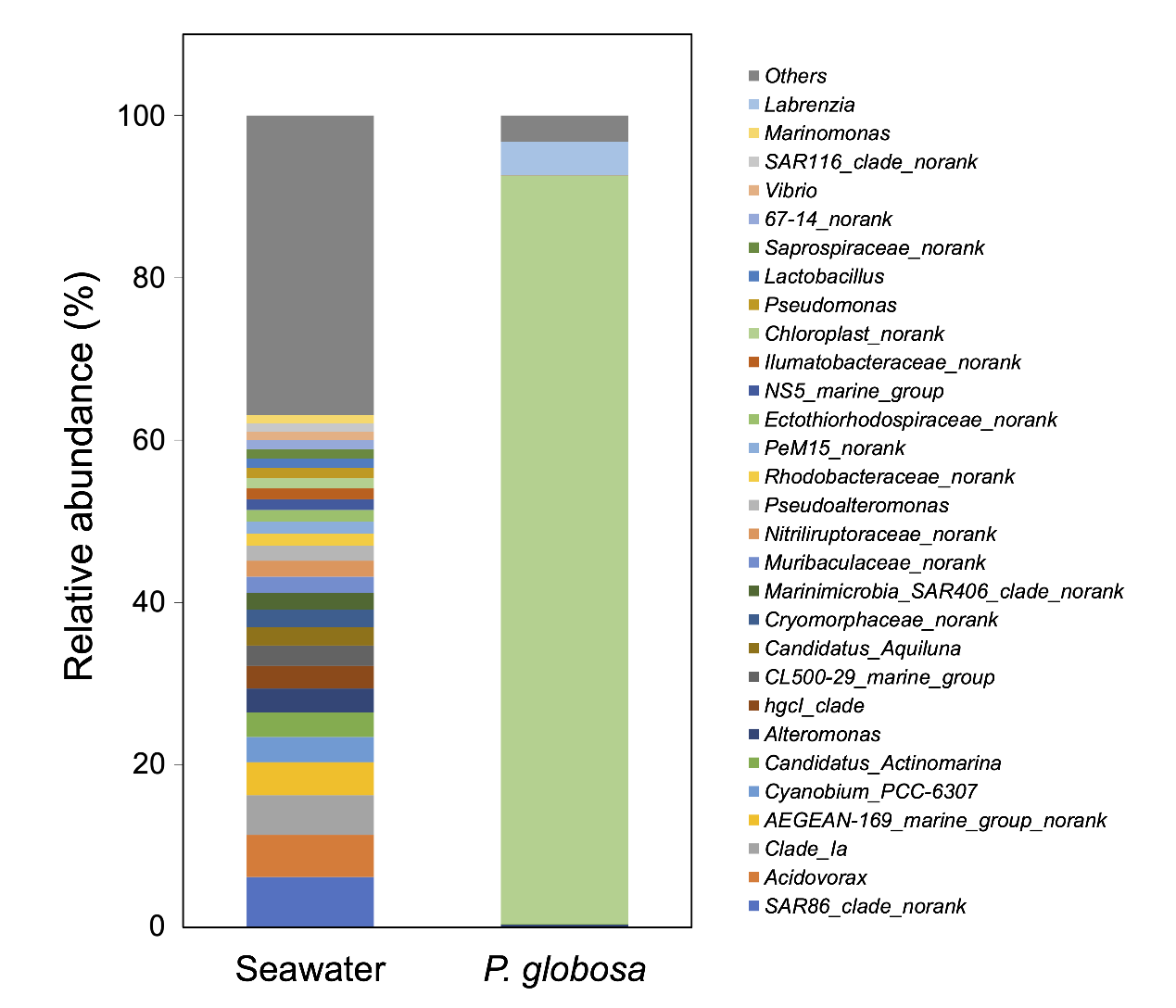


**Supplementary Figure 1** Microbial composition of ambient seawater and *Phaeocystis globosa* intracolonial fluid identified by primer pairs 338F-806R. The stacked bar plots show the relative abundance of OTUs at genus levels. Each bar represents the average of pooled replicates.


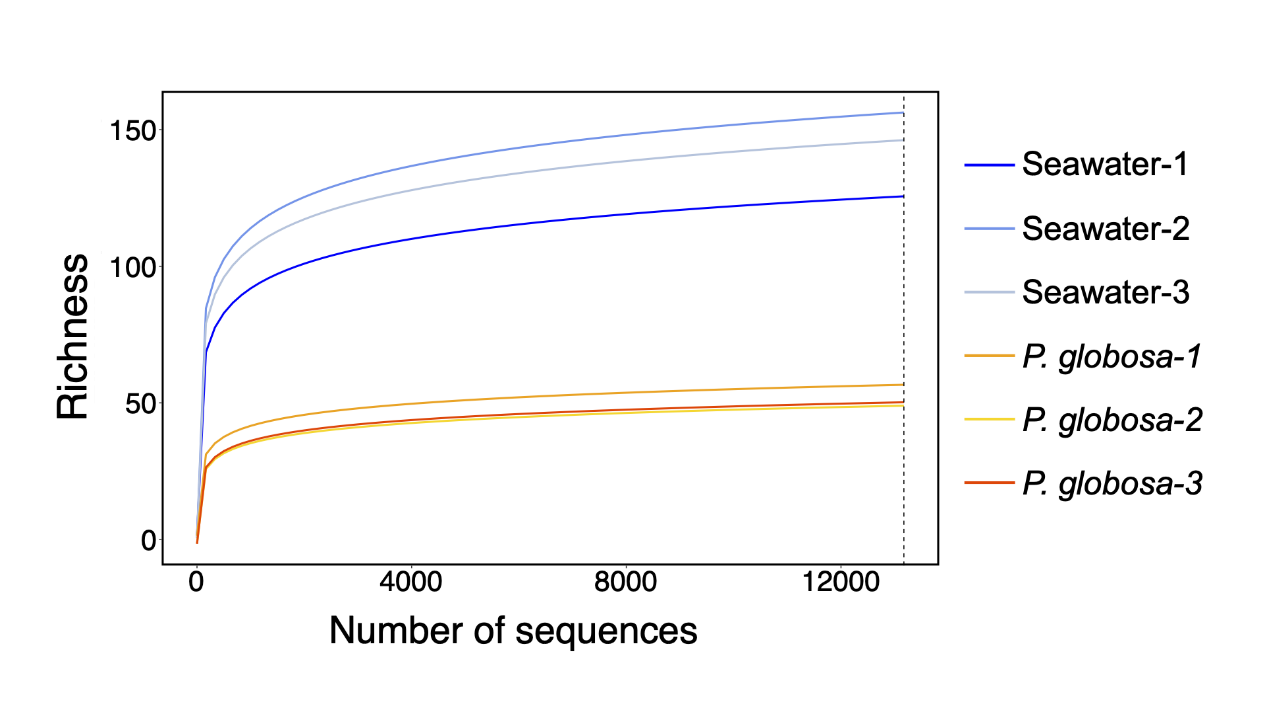


**Supplementary Figure 2** Rarefaction curve of the samples from ambient seawater and *Phaeocystis globosa* intracolonial fluid. Each curve represents an individual sample.


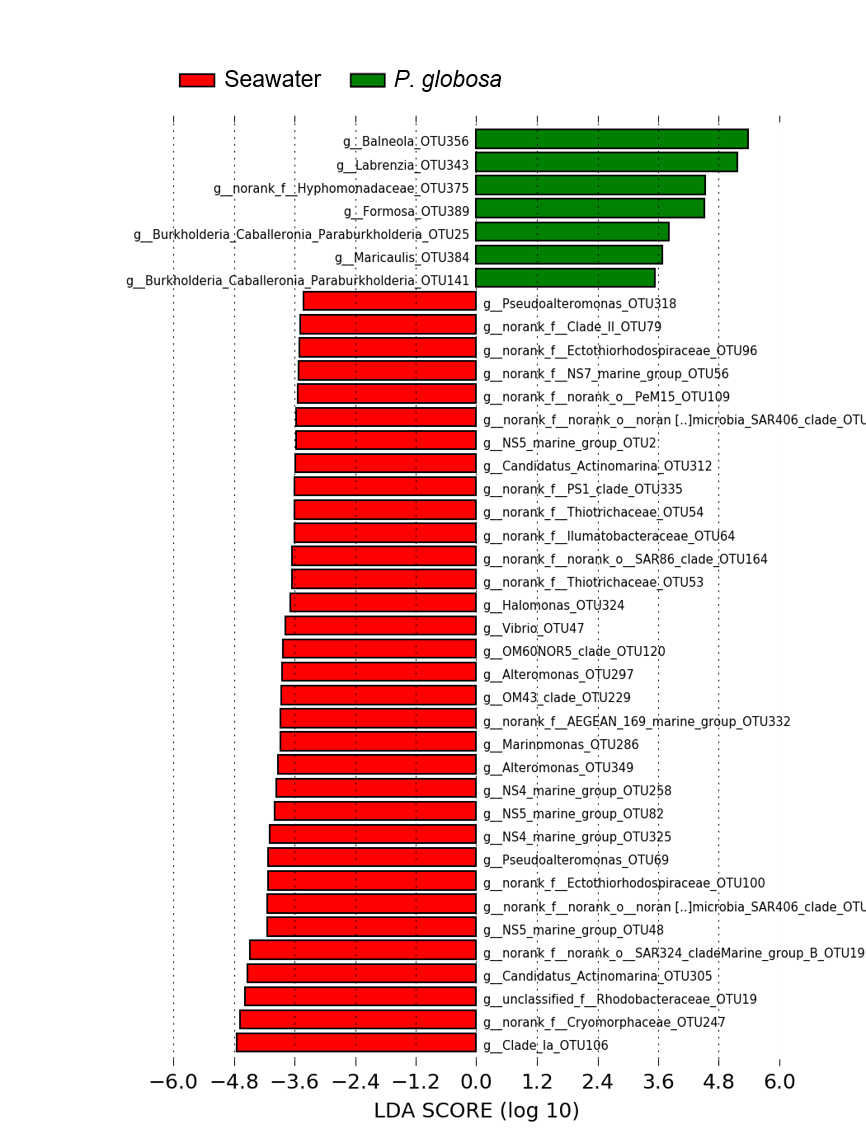


**Supplementary Figure 3** LEfSe analysis was used at OTU level to determine the dominant species between ambient seawater and *Phaeocystis globosa* intracolonial fluid.

**Supplementary Table 1** OTU identification and total number of reads associated to each OTU per sample.

**Supplementary Table 2 The Nearest Sequenced Taxon Index (NSTI) scores of each sample.**

**Supplementary Table 3** KEGG pathways identification in each seawater samples and *Phaeocystis globosa* intracolonial fluid samples.
